# Prevalence and Genetic Structures of *Streptococcus pneumoniae* Serotype 6D, South Korea

**DOI:** 10.3201/eid1611.100941

**Published:** 2010-11

**Authors:** Eun Hwa Choi, Hoan Jong Lee, Eun Young Cho, Chi Eun Oh, Byung Wook Eun, Jina Lee, Min Ja Kim

**Affiliations:** Author affiliations: Seoul National University Children’s Hospital, Seoul, South Korea (E.H. Choi, H.J. Lee, E.Y. Cho, C.E. Oh, B.W. Eun);; Seoul National University College of Medicine, Seoul (E.H. Choi, H.J. Lee);; Kosin University College of Medicine, Busan, South Korea (C.E. Oh);; Gachon University Gil Hospital, Incheon, South Korea (B.W. Eun);; Seoul National University Bundang Hospital, Seongnam, South Korea (J. Lee);; Korea University College of Medicine, Seoul (M.J. Kim)

**Keywords:** Streptococcus pneumoniae, serotype, epidemiology, bacteria, dispatch

## Abstract

To determine prevalence and genetic structures of new serotype 6D strains of pneumococci, we examined isolates from diverse clinical specimens in South Korea during 1991–2008. Fourteen serotype 6D strains accounted for 10.4% of serogroup 6 pneumococci from blood, sputum, nasopharynx, and throat samples. Serotype 6D strains consisted of 3 sequence types.

*Streptococcus pneumoniae* is a common cause of invasive infection in infants, children, and adults. The polysaccharide capsule of *S. pneumoniae* is the major virulence factor that protects the organism from host phagocytosis (*1*). Recently, 2 new serotypes of serogroup 6 pneumococci, 6C and 6D, were genetically and biochemically characterized (*2**,**3*). Serotype 6C was identified in 2007 on the basis of its distinct binding patterns with 2 monoclonal antibodies; serotype 6C had previously been typed as 6A according to the standard quellung reaction. Serotype 6C produces glucose in the place of galactose in the 6A capsular polysaccharide and has the *wciN_β_* gene, which is ≈200 bp shorter than the corresponding *wciN* gene in 6A (*2**,**4*). After the discovery and characterization of 6C through genetic and biochemical studies, a new experimental serotype, 6X1 (later named 6D), was created by mutating the critical nucleotide in the *wciP* gene of the 6C capsule gene locus or by inserting the *wciN_β_* gene into the 6B capsule gene locus (*3*). However, this putative serotype, 6D was thought to not exist in nature until recently, when 2 studies found 6D strains in nasopharyngeal aspirates from children in Fiji during 2004–2007 (*5*) and in 2 nasopharyngeal aspirates from children in South Korea in 2008 (*6*). Although serotype 6C has only recently been described, several studies indicate that serotype 6C pneumococci have been circulating in many countries, including the United States, the Netherlands, Australia, Israel, and South Africa (*7**–**10*). However, reports of naturally occurring serotype 6D pneumococci are limited.

We investigated the prevalence of serotypes 6C and 6D in 2 collections of pneumococci isolated from clinical specimens in South Korea. We compared the genetic diversity and antimicrobial drug susceptibility patterns of the 4 serotypes, 6A, 6B, 6C, and 6D.

## The Study

Of the 2 collections of pneumococcal isolates, the first consisted of 587 clinical specimens obtained from infants and children at Seoul National University Children’s Hospital, Seoul, South Korea, from May 1991 through May 2008. The second collection consisted of 225 clinical specimens obtained from adults at 2 participating hospitals in Seoul from March 2004 through August 2007. When >1 isolate was recovered from the same person, only the initial isolate was included in the study. From these 2 sample collections (n = 812), we redetermined serotypes for 134 isolates previously assigned to serogroup 6.

Serotyping was performed by using the quellung reaction with antiserum for serogroup 6, factor 6b, and factor 6c (Statens Serum Institute, Copenhagen, Denmark). To assign serotypes 6C and 6D, we screened all strains for *wciN_β_* and *wciP_6B_* by using 2 simplex PCRs and subsequent sequencing analysis. The *wciN* gene was amplified with the forward primer (5106) 5′-TAC CAT GCA GGG TGG AAT GT-3′ and the reverse primer (3101) 5′-CCA TCC TTC GAG TAT TGC-3′, resulting in product sizes of 1.8 kb for serotypes 6C and 6D for the *wciN_β_* gene (*2*). The *wciP* gene was amplified by using the forward primer 5′-AAT TTG TAT TTT ATT CAT GCC TAT ATC TGG -3′ and the reverse primer 5′-TTA GCG GAG ATA ATT TAA AAT GAT GAC TA-3′ (*11*). Presence of *wciN_β_* and *wciP_6B_* was confirmed by sequencing analysis. A characteristic of 6B *wciP* is the presence of an A at nucleotide position 584 (according to the sequence of *wciP* [12]), which creates a codon for asparagine at residue 195 of the 6B *wciP* protein. Antimicrobial drug susceptibility testing, multilocus sequence typing (MLST), and eBURST analyses were performed as described (*13*).

Capsular swelling reactions indicated 63 serotype 6A and 61 serotype 6B strains. However, 10 strains were not distinguished by the standard method (quellung reaction) because they reacted with both factors, 6b and 6c. Sequencing analysis showed that 6 serotype 6A strains were serotype 6C according to the presence of *wciN_β_* but the absence of *wciP_6B_*. Subsequently, 4 serotype 6B strains and 10 undistinguished strains were identified as serotype 6D on the basis of the presence of *wciN_β_* and *wciP_6B_*.

Serotypes tested by using the molecular method were 6A (n = 53, 39.6%), 6B (n = 61, 45.5%), 6C (n = 6, 4.5%), and 6D (n = 14, 10.4%). The earliest recovery of a serotype 6D isolate was in 1996, and the earliest recovery of a serotype 6C isolate was in 1993. Two serotype 6D strains were obtained from adults, and the remaining 12 strains were obtained from infants or children. Sources of serotype 6D isolates were blood (n = 5), sputum (n = 6), nasopharynx (n = 2), and throat (n = 1) specimens (Table). All serogroup 6 isolates except a 6C strain showed multidrug resistance to at least 3 classes of antimicrobial drugs. According to MLST, 3 sequence types (STs) were found in serotype 6D pneumococci (ST189 [n = 7], ST3171 [n = 4], and ST282 [n = 3]), which fell into 2 clonal complexes according to eBURST analysis (Figure). ST189 and ST282 were closely related to clonal complex 81, which clustered with serotype 6A strains. All 4 ST3171 strains were isolated from blood. Each ST exhibited distinct antimicrobial drug susceptibility patterns and genes for macrolide resistance (Table).

**Table:** Genetic structures and phenotypes of 14 strains of *Streptococcus pneumoniae* serotype 6D, Seoul, South Korea*

CC or ST and year of isolation	Patient age	Sample source	MIC, μg/mL			Macrolide resistance gene	
			Penicillin	Cefotaxime		*mef*A	*erm*B
CC81							
ST189							
2000	5 y	Throat swab	1.50	0.75		Present	Absent
2004	81 y	Sputum	2.00	1.00		Present	Absent
2006	1 y	Sputum	1.50	1.00		Present	Absent
2006	2 y	Sputum	1.00	0.75		Present	Absent
2007	5 y	Sputum	1.50	0.75		Present	Absent
2007	6 y	Nasopharynx	1.50	0.75		Present	Absent
2007	73 y	Blood	2.00	1.00		Present	Absent
ST282							
2004	4 mo	Sputum	1.50	0.75		Present	Absent
2005	8 mo	Nasopharynx	1.50	0.75		Present	Absent
2005	9 mo	Sputum	1.50	0.75		Present	Absent
ST3171							
1996	1 y	Blood	0.06	0.05		Absent	Present
1997	3 y	Blood	0.06	0.50		Absent	Present
1997	14 y	Blood	0.06	0.50		Absent	Present
1997	15 y	Blood	0.06	0.50		Absent	Present

*CC, clonal complex; ST, sequence type. Antimicrobial drug susceptibility testing, detection of *mef*A/*erm*B, multilocus sequence typing, and eBURST analyses were performed as described (*13*). All strains were resistant to at least 3 antimicrobial drug classes.

**Figure:**
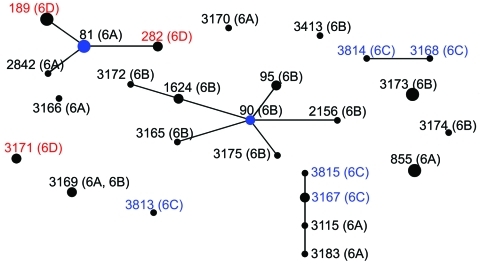
eBURST analysis of 134 strains of *Streptococcus pneumoniae* serogroup 6. Serotypes are indicated in parentheses. Circle size correlates with number of strains of each sequence type. Blue circles indicate predicted founders (original sequence types within the cluster). Serotype 6D is shown in red, serotype 6C in blue.

## Conclusions

We identified 14 naturally occurring serotype 6D strains among 134 serogroup 6 pneumococci collected from diverse clinical specimens in South Korea during 1991–2008. The prevalence rate of serotype 6D among serogroup 6 isolates was 10.4%, slightly higher than that of serotype 6C (4.5%). Although serotype 6D was only recently discovered, we demonstrated that serotype 6D strains have been circulating since at least 1996. Serotype 6D was identified from various clinical sources, including blood, sputum, throat swab, and nasopharynx specimens, contrasting with findings of 2 previous studies (*5**,**6*).

The genetic structures of serotype 6D pneumococci in the MLST database (www.mlst.net) were single isolates of ST4241 (Australia); ST982, ST4190, ST5085, and ST5086 (China); and 2 isolates of ST282 (South Korea). Of those, 3 strains from China (ST982, ST5085, and ST5086) were closely related to the ST3171 strain from South Korea. This cluster of serotype 6D strains was associated with serotype 6A and 6B isolates from 3 countries in Asia. A single isolate of ST4241 was related to STs associated mostly with serotype 6B, but the ST4170 strain did not seem to be linked to other STs. This study demonstrated that 7 serotype 6D strains of ST189 and 3 serotype 6D strains of ST282 were related to clonal complex 81, which had previously been associated with only serotype 6A isolated from South Korea. However, this clonal complex also included several STs associated with many other global serotypes, such as 23F, 19F, and 19A. Although the mechanism is not completely clear, available data indicate that capsular switching from serotypes 6A, 23F, 19F, or 19A to serotype 6D is possible; this switching could occur in addition to replacement of the *wciN_β_* gene into the 6B capsule gene locus. A previous study indicated capsular switching as the possible event for formation of serotype 6C isolates (*14*).

In a recent study, factor 6d antiserum was validated for accurate serotyping of 6C (*10*) and is now commercially available, but antiserum for detection of 6D has not yet been developed. Further studies will be required to investigate the prevalence and genetic relatedness of serotype 6D pneumococci in different countries and to evaluate the effect of pneumococcal conjugate vaccine on serotype distribution.
